# Supplementary material for: Abundance and Distribution of Phlebotomus pedifer (Diptera: Psychodidae) Across Various Habitat Types in Endemic Foci of Cutaneous Leishmaniasis in the Mid-Highlands of Wolaita Zone, Southern Ethiopia
Source: Trop Med Infect Dis. 2024 Dec 10;9(12):302. doi: 10.3390/tropicalmed9120302 (PMC11679830; doi:10.3390/tropicalmed9120302)
Supplement: Supplementary file 1 [file tropicalmed-09-00302-s001.zip › tropicalmed-3291717-supplementary.pdf]

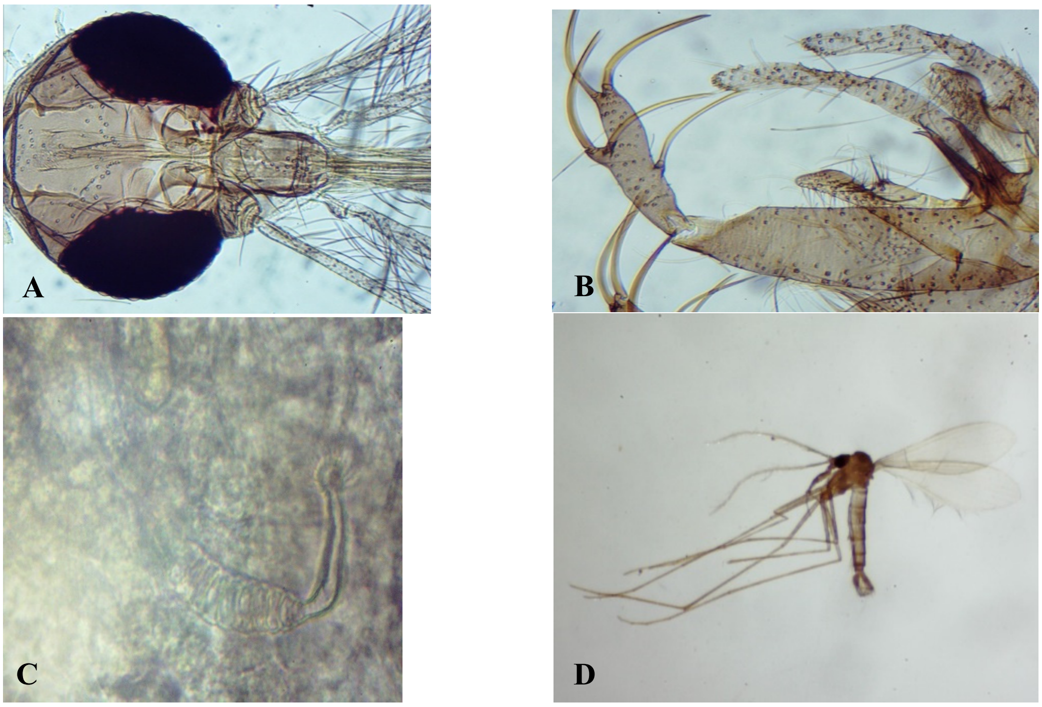

Figure S1: Representative images of *P. pedifer* morphological identification: (A) head part (10X magnification); (B) last abdominal segment showing the male genitalia (10X); (C) spermathecae (40X); (D) undissected male (4X). The images were captured using a Swiftcam eyepiece camera attached to an Olympus compound light microscope.
